# Safety and efficacy of Vectored thermal pulsation, Intense pulsed light, and Eyelid Warm compress therapies (VIEW) for meibomian gland dysfunction: Study design and baseline characteristics of a randomized controlled trial

**DOI:** 10.1371/journal.pone.0342421

**Published:** 2026-04-02

**Authors:** Zhichao Hu, Hanson Yiu Man Wong, Emily Hoi Ying Chan, Eric Ka Ho Choy, Andy Chak Fung Ng, Xulin Liao, Fatema Mohamed Ali Abdulla Aljufairi, Jake Uy Sebastian, Kenneth Ka Hei Lai, Charlotte Yi Sum Poon, Wanxue Chen, Vanissa Wing See Chow, Clement Chee Yung Tham, Chi Pui Pang, Kelvin Kam Lung Chong

**Affiliations:** 1 Department of Ophthalmology and Visual Sciences, Faculty of Medicine, The Chinese University of Hong Kong, Hong Kong, China; 2 Department of Ophthalmology, Salmaniya Medical Complex, Government Hospitals, Manama, Bahrain; 3 Department of Ophthalmology, Vicente Sotto Memorial Medical Center, Cebu, Philippines; 4 Department of Ophthalmology and Visual Sciences, Princes of Wales Hospital, Hong Kong, China; 5 Hong Kong Eye Hospital, Hong Kong, China; 6 Eye Centre, The Chinese University of Hong Kong Medical Centre, Hong Kong, China; Boston University School of Medicine, UNITED STATES OF AMERICA

## Abstract

**Background:**

Meibomian gland dysfunction (MGD) is one of the most prevalent chronic eye diseases, characterized by meibomian gland obstruction and abnormal meibum secretion both in quantity and quality.

**Objective:**

To assess the efficacy and safety of vectored thermal pulsation (VTP), intense pulsed light plus meibomian gland expression (IPL + MGX), and eyelid warm compress (EW).

**Methods:**

This is a 3-arm, assessor-masked, randomized controlled trial. 360 symptomatic mild to moderate MGD subjects would be recruited and randomized into 3 intervention groups. Subjects from Group-A, Group-B, and Group-C would be treated with one-session VTP (at baseline), four-session IPL + MGX (at baseline, month-1, month-2, and month-3 visits), and twice-daily EW for 15 months, respectively. All subjects would be prescribed topical artificial tears (Hypromellose, 0.3% w/v) to be used as frequently as needed from wash-out (1 month before baseline) to endpoint (month-15 visit). Subjects would be assessed 9 times throughout a total of 16-month study period. Five self-administered questionnaires would be collected each time in random order. Masked observers would conduct examinations using slit-lamp biomicroscopy, LipiView® II, and Keratograph 5M machines. The eye with worse MGD would be selected and analyzed as the study eye. Tear film break-up time (TFBUT) at month-6 and month-15 visits are the primary outcomes.

**Results:**

374 subjects were enrolled and randomized into three groups, and 372 subjects (aged 54.4 ± 14.7 years) were finally analyzed. The mean of TFBUT time at baseline was 3.1 ± 1.7 seconds.

**Conclusion:**

This is the first head-to-head comparison of three physical treatments for mild to moderate MGD on their therapeutic efficacy, safety, longevity and compliance in a randomized controlled trial setting.

## Introduction

Meibomian gland dysfunction (MGD) is a chronic and progressive condition characterized by terminal duct obstruction with qualitative and/or quantitative changes in meibum secretion [1]. MGD affects approximately 30-40% of the global population [2]. The prevalence in the Asia Pacific region is especially high, up to 60%. However, the true prevalence remains elusive as there is a lack of consensus on objective and subjective measurements to diagnose and grade MGD. MGD is closely related to and shares overlapping symptoms and signs with evaporative subtype of dry eye disease or blepharitis. Disturbance to meibum secretion and/or distribution will cause tear film instability, increased tear evaporation and osmolarity, triggering ocular surface inflammation, epithelial damage, and a vicious cycle of ocular surface disease [3].

At present, treatments for MGD start with self-administered therapies to prescription medications, to slow down progression, provide symptomatic relief without curative intent. Artificial tears or topical lubricants, eyelid warm compress therapy (EW), performing eyelid hygiene, have all shown to have limited efficacy and suboptimal compliance in moderate-to-severe MGD patients [4]. While topical steroids, antibiotics, and immunomodulatory agents may show better short-term effects [5–7], concerns were raised regarding adverse reactions including antibiotic resistance, steroid-related cataract and ocular hypertension. Long-term use of these prescription medications is also limited by costs, accessibility, and the need for regular ophthalmology monitoring [8,9]. Therefore despite many available treatment options, management of MGD is often considered unsatisfactory and frustrating by clinicians and patients [10].

Among emerging treatment options, the intense pulsed light (IPL) therapy has gained popularity in recent years. With good efficacy and safety, IPL has been widely adopted for both cosmetic and therapeutic indications in a wide range of dermatological conditions. Incidental correlations have been made between the therapeutic effect of IPL and MGD when rosacea patients undergoing facial IPL experienced concurrent MGD improvements [11]. This has led to combining IPL and meibomian gland expression (MGX) as multi-session practitioner-administered physical therapy for MGD. Multiple reviews and meta-analyses have confirmed its efficacy and safety, yet the longevity of IPL and MGX beyond 6 months after the last treatment was questioned [12–17].

Vectored thermal pulsation (VTP) is FDA-approved treatment for MGD as LipiFlow® system [18]. Covering both the cutaneous and mucosal surfaces of both eyelids, the device transmits heat to the MG, melts the meibum, massages the meibomian glands and their openings aiming to resume the normal meibum flow. According to a meta-analysis, the efficacy of one single 12-minute session VTP was better than EW in treating MGD [18].

Currently, there is a lack of level I evidence comparing the efficacy between the two practitioner-administered therapies, single-session VTP and multi-session IPL + MGX, with home-based, self-administered twice-daily EW. Importantly, the onset and offset of therapeutic effects, time course of single-session VTP, multi-session IPL + MGX, or twice-daily EW on MGD up to 12 months post last IPL treatment have never been studied in an RCT setting. Therefore, this study aims to: 1) compare the efficacy and safety of one-session VTP, four-session IPL + MGX with EW therapy for mild to moderate MGD; and 2) compare the course of MGD among groups over 15 months; and 3) identify factors predicting therapeutic responses and compliance.

## Methods

### Design

**Fig 1:**
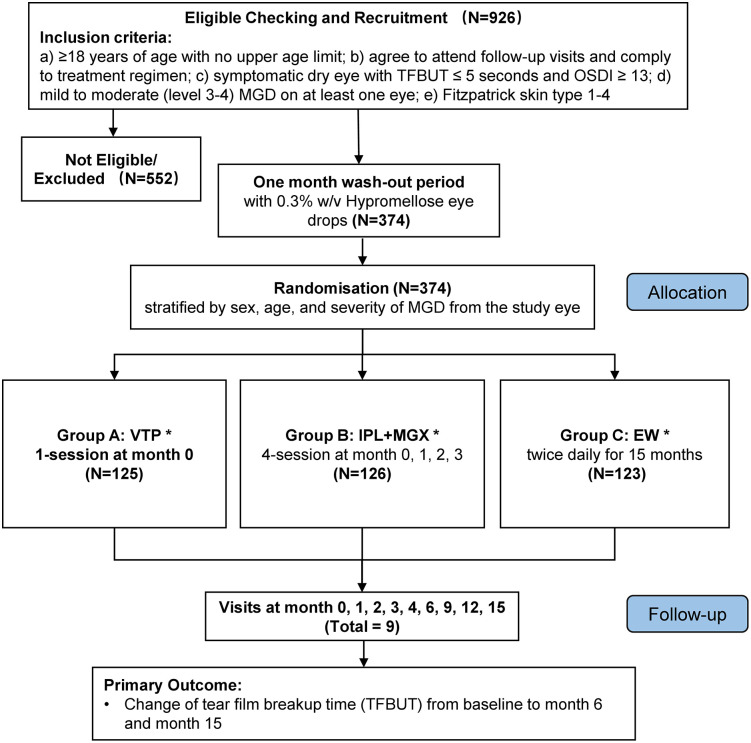
Flow diagram of the study design, randomization, and treatment. VTP: vectored thermal pulsation; IPL: intense pulsed light; MGX: meibomian gland expression; EW: eyelid warm compress; *: topical artificial tears (Hypromellose, 0.3% w/v) were provided from wash-out to month 15 (exit).

This 3-arm, assessor-masked randomized controlled trial was conducted at the Chinese University of Hong Kong Eye Centre (CUHKEC) and Eye Centre, CUHK Medical Centre (CUHKMC). Patients with mild to moderate MGD were referred from the participating hospitals and by open recruitment, coordinated by the Department of Ophthalmology and Visual Science (DOVS) of CUHK. Subjects were randomized and allocated to Group A for VTP, Group B for IPL + MGX, or Group C for EW. The study flow diagram detailing the randomization, treatment and follow-up is shown in Fig 1. The recruitment started on 1st October 2022 and finished on 3rd March 2024 (17 months). The data collection was completed on 10th August 2025, and the primary outcomes are expected to be available in Q2/Q3 2026.

The inclusion and exclusion criteria were developed with reference to the recent RCTs on MGD [19,20]. Inclusion criteria include: 1) ≥18 years of age with no upper age limit; 2) Agree to attend follow-up visits and comply with the treatment regimen; 3) Symptomatic dry eye with tear film break-up time (TFBUT) ≤ 5 seconds and the Ocular Surface Disease Index (OSDI) ≥13 [21,22]; 4) Mild to moderate (level 3–4) MGD in at least one eye [23]; 5) Fitzpatrick skin type I-IV.

The subjects who fulfill any of the following exclusion criteria were excluded from the study: 1) Use of contact lenses within 3 months before or during the study period; 2) Use of topical (including topical anti-glaucomatous, cyclosporin, antibiotics) or systemic medications known to worsen or improve MGD within 3 months before or during the study period; 3) Major systemic illnesses (e.g. Sjogren’s syndrome), ocular conditions (including thyroid eye disease, recurrent conjunctivitis, ocular allergies), or dermatological diseases (e.g., rosacea) known to affect MGD; 4) Undergoing ocular procedures (excluding uncomplicated cataract operation) within 3 months before or during the study period; 5) History of vision correction surgery or plan to undergo such procedure during the study period; 6) Receiving dermatological treatment (including chemical peeling, laser, IPL or energy device in the periocular and facial region) within 6 months before or during the study period; 7) Contraindications to IPL therapy (including recent sun-burn, photosensitivity, active or pigmented skin lesions, cancer, implants, tattoos, semi-permanent makeup in the periocular area); 8) Contraindications to VTP therapy (ocular surgery, ocular injury, ocular herpes, ocular inflammation, active ocular infection within 3 months before the start of the study); 9) Female subjects who are pregnant, nursing, planning pregnancy, or of childbearing potential and not using a reliable method of contraception.

### Sample size calculation

The primary outcome of this study, TFBUT, is selected for the sample size calculation. According to previous studies, the estimated mean differences in changes of TFBUT are 3.3s for IPL + MGX and 1.9s for VTP, and the pool estimates of standard deviations (SDs) are 1.88 for IPL + MGX and 4.04 for VTP [24,25]. Based on a two-sided α of 0.025 after Bonferroni correction, an assumed average SD of 3, and 80% statistical power, a minimum sample of 88 patients per group was required to detect a significant difference of TFBUT. Allowing for a 25% dropout rate over the 16-month period from wash-out to study exit, 120 subjects per group were required. This sample size is large enough to detect differences between VTP and EW or between IPL + MGX and EW.

### Randomization and allocation concealment

A computer-driven minimization program was applied for the randomization procedure by research staff not involved in the rest of the study [26]. Minimization, dynamically randomizes subjects into different groups, ensuring good inter-group balance. Sex, age, and severity of MGD were selected as predefined covariate factors in this RCT. Study personnel responsible for the outcome measurements were masked. The subjects and treating investigators conducting the VTP or IPL + MGX procedures were not masked. All subjects remained in their assigned intervention groups during the entire study period.

### Interventions

#### VTP procedure.

Single-session VTP therapy was delivered by the LipiFlow® Thermal Pulsation System (Johnson & Johnson, Jacksonville, FL, USA) at the baseline visit. The thermal pulsation system device, with a pair of disposable activators for each subject, is automated and computer-controlled. The activator provides a constant temperature of 42.5°C and simultaneously evacuate both upper and lower eyelid MG with gentle pressure (maximum 6 psi). One to two drops of topical anesthesia (Proparacaine Hydrochloride, 0.5% w/v) would be instilled into both eyes respectively before placing the activators. This 12-minute treatment was performed by the unmasked treating investigators. Procedure-related pain scores by the Visual Analogue Scale (VAS) would be obtained for each part (application of activators, treatment cycle, and overall procedure) by the treating investigators immediately.

#### IPL and MGX procedure.

Four-session IPL (baseline, month-1, month-2, and month-3 visit) was administered using the Lumenis® M22 (Lumenis, Borehamwood, UK) according to the Toyos protocol [11]. The proprietary “dry eye mode” setting of the machine was applied. Pulse intensity (11–14 J/cm^2^), delivered via a 560 nm filter, was chosen based on inverse relations to the Fitzpatrick skin phototype of each subject. Adjustments of pulse intensity were be made based on subjects’ pain tolerance after the pre-treatment test pulse delivered to the skin of each subject's inner wrist/arm or treatment-related pain during/after each IPL treatment. A similar energy level was used for each subject throughout the four sessions.

Before the IPL procedure, both eyelids would be sealed with IPL-aid disposable shields for eye protection. Cool conducting gel would be applied evenly on the treatment area (inferior and temporal portions of both eyes). All subjects received a total of 10 IPL pulses in each eye in each session. 4 IPL pulses would be delivered to four overlapping zones inferior to each eye, with 1 extra pulse delivered temporal to the lateral canthus. After the first round was completed, the second cycle was applied respectively to the inferior and temporal area in each eye again. Safety goggles were worn by the unmasked treating investigator during the IPL procedures.

MGX would be immediately performed on both the upper and lower eyelids of each eye under the slit-lamp biomicroscope using a pair of sterile stainless-steel MG expressor forceps for expression with appropriate force. Topical anesthetic eye drops (Proparacaine Hydrochloride, 0.5% w/v) would be applied on both eyes before MGX to minimize the pain. Each part of procedure-related pain score (IPL, MGX, and overall procedure) by VAS would be obtained immediately.

#### EW procedure.

A warm and wet towel would be placed over the eyes twice daily at home by subjects during the follow-up period (15 months) [8,9]. Each EW session lasts for around 10 minutes. A standard educational material with detailed instructions and precautions of EW were given to subjects to ensure the procedures were conducted consistently. Subjects were also requested to mark the treatment record in a standard diary provided. Compliance was monitored through the treatment diaries and reviewed by the study team at each visit. Participants were not excluded for poor compliance; however, compliance was encouraged at each visit and will be analyzed as an exploratory covariate.

#### Topical lubricant.

All subjects were given the same topical lubricant (Hypromellose, 0.3% w/v) to be used as frequently as needed during the wash-out (1 month before baseline) and throughout the entire follow-up (15 months) period.

### Primary outcome

The primary outcome is the change of TFBUT from baseline to month-6, as well as from baseline to month-15 visit. The TFBUT is the time for the initial break-up of the tear film after a blink. A drop (2 µL) of 2% sodium fluorescein solution would first be instilled into each lower eyelid of the subject using a pipette tip. A follow-up investigator would then conduct the examination by the slit-lamp with a broad beam of cobalt blue light, and record the time between full blinking and the appearance of the first dry spot or hole using the stopwatch function of a mobile phone. The test was repeated 3 times in the study eye first, and the average of TFBUT was taken before staining and measuring the fellow non-study eye. To minimize variability in TFBUT measurements, all subjects were scheduled for the same session at each visit, and all measurements were carried out between 9:00 and 17:00. The time of examination was marked on the case report form.

### Secondary outcomes

#### Questionnaires.

Demographic and baseline characteristics, medical and ophthalmic conditions, surgeries and hospitalization history, smoking & alcohol use, lifestyle, and work productivity were collected during recruitment. Five self-administered questionnaires in traditional Chinese would be performed in random order at each visit: the OSDI [21], Standard Patient Evaluation of Eye Dryness (SPEED) [27], Symptom Assessment iN Dry Eye (SANDE) [28], Ocular Comfort Index in Chinese (OCI-C) [29], Dry Eye Questionnaire (DEQ-5) [30].

#### LipiView® II Ocular Surface Interferometer.

Infrared images of lower and upper eyelid meibomian gland structures were captured by the LipiView® II Ocular Surface Interferometer (TearScience, Morrisville, NC). Meiboscore and meibograde were obtained for the quantitative and qualitative assessment of gland structure abnormalities. Meiboscore is a four-point grading scale considering the ratio of MG dropout over total analyzed MG area (0: no dropout of MG; 1: with less than 1/3 of MG area dropout; 2: with 1/3–2/3 of MG area dropout; 3: more than 2/3 of MG area dropout) [31]. Meibograde consists of 3 grading parts in terms of morphological changes of MG: distortion, dropout, and shortening, with each of these characteristics graded from 0–3 [32]. Partial blinking rate and lipid layer thickness (LLT) would be automatically calculated and reported by the machine. The modified Guillon-Keeler system would be used to grade the tear interferometric fringe pattern [33].

#### Keratograph 5M.

Keratograph 5M (KG 5M) corneal topographer (OCULUS, Wetzlar, Germany) is a non-invasive device with a built-in keratometer and color camera. Non-invasive keratographic break-up time (NIKBUT), bulbar conjunctival hyperaemia, and lower eyelid tear meniscus height (TMH) would be collected by the KG 5M. The machine would measure NIKBUT 3 times and the average value was taken. The TMH would be calculated as the average height of three measurement points (nasal, temporal limbus, and pupil centre) of the lower meniscus. Bulbar conjunctival hyperaemia was automatically graded and reported by the JENVIS Pro Dry Eye Software (OCULUS, Wetzlar, Germany).

#### Other slit-lamp biomicroscope examinations.

Corneal and conjunctival epithelial surface damage was evaluated by the Oxford grading system after staining by 2% w/v sodium fluorescein. The severity was graded on a six-point Oxford scheme from 0 to 5 based on the number of dots at the corneal and conjunctival regions [34,35]. Lid wiper epitheliopathy (LWE) was graded by the linear area and severity of staining [36].

Five MGs at the temporal, nasal, and central areas respectively of each lower eyelid (total 15 glands) were selected for the expressibility assessment. The glands’ meibum secretion quality was graded on a four-point scale (0: no secretion; 1: inspissated or toothpaste-like liquid secretion; 2: cloudy liquid secretion; 3: clear liquid secretion) with a sum of 0–45 [18]. The number of glands that yielded clear meibum at the temporal, nasal, and central areas of the low eyelid would be counted as the meibomian glands yielding liquid secretion (MGYLS) [37].

Lid margin and eyelash abnormalities (thickening, rounding, notching, foaming, telangiectasia, capping, staphylococcal crusting, seborrheic crusting, Demodex cylindrical collarettes, madarosis, poliosis, and trichiasis) were also graded on a four-point scale (0: absent; 1: mild; 2: moderate; 3: severe).

#### Schirmer test.

The standard sterile Schirmer paper strip (5 mm × 35 mm) would be gently placed onto each lower fornix as near as possible to the lateral canthus without anesthesia by the investigator. Subjects would be required to close their eyes for 5 minutes. Afterwards, the investigator would mark down the length of wet portions (millimeters) according to the marking scale. The strips would be placed into sterile centrifuge tubes and stored at −80°C for further laboratory tests.

#### Specimen Collection.

Tear samples were collected on the standard sterile filter paper strip during the Schirmer test (ST) and stored in the 2 mL sterile microcentrifuge tubes at each follow-up visit. Tears laboratory analyses would focus on inflammatory biomarkers. Subjects’ blood samples were collected at baseline for potential biomarker studies. Subjects’ conjunctival microbiome samples were collected by a sterile swab on the inferior fornix of the conjunctiva at baseline, month-4 visit and endpoint and stored in 2 mL sterile microcentrifuge tubes for further metagenomic sequencing. The change of diversity before and after treatment would be analyzed and reported [38].

The first type of meibum sample was collected from the lower eyelids of Group B subjects by a sterile swab after the MGX procedure immediately at baseline, month-1, month-2, and month-3 visits, and stored in 2 mL microcentrifuge tubes. The second type of meibum sample would be collected on the lower eyelids of all subjects by a sterile swab during MG expressibility assessment at baseline, month-4 visit, and endpoint, and stored in 2 mL microcentrifuge tubes. Samples collected were obtained using sterile swabs without touching the eyelid skin.

All samples were collected using sterile equipment following standard operating procedures (SOPs) designed to minimize contamination. All tears, conjunctival swab, and meibum samples were sealed immediately after collection. After corresponding preprocessing, the samples would be transferred to a −80°C laboratory freezer for long-term storage and further laboratory tests.

### Adverse events and safety assessments

All adverse events were recorded by masked trial investigators for each subject. Examinations, including anterior chamber activities, dilated fundus examination, lens opacities, and iris defect and transillumination under slit-lamp biomicroscope, were conducted to assess the safety in each subject. Anterior chamber activities were graded by the Standardization of Uveitis Nomenclature (SUN) grading system [39]. The lens opacities were graded by the Lens Opacities Classification System (LOCS II) [40]. A dilated fundus examination was conducted to evaluate potential abnormality of the macula and optic disc. Loss of lashes and eyebrows, facial redness, swelling, bruises, and pigmentation would be recorded.

The same order of tests and procedures, from least to most invasive, was followed at each visit. An overview of procedure and examination at each visit is shown in Table 1.

**Table 1:** The Overview of Procedure and Examination at Each Visit.

Assessments/ number of month	Screening	V1	V2	V3	V4	V5	V6	V7	V8	V9
	–	M0	M1	M2	M3	M4	M6	M9	M12	M15
**Procedure**										
Consent	Y									
Eligibility checking	Y									
Wash-out (M −1)	Y									
**EFFICACY (SUBJECTIVE)**										
Ocular Surface Disease Index (OSDI)	Y	Y	Y	Y	Y	Y	Y	Y	Y	Y
Standardized Patient Evaluation of Eye Dryness (SPEED)		Y	Y	Y	Y	Y	Y	Y	Y	Y
Symptom Assessment iN Dry Eye (SANDE)		Y	Y	Y	Y	Y	Y	Y	Y	Y
Ocular Comfort Index in Chinese (OCI-C)		Y	Y	Y	Y	Y	Y	Y	Y	Y
Dry Eye Questionnaire (DEQ-5)		Y	Y	Y	Y	Y	Y	Y	Y	Y
**EFFICACY (OBJECTIVE)**										
Non-invasive keratograph break-up time (NIKBUT)		Y	Y	Y	Y	Y	Y	Y	Y	Y
Conjunctival bulbar hyperaemia		Y	Y	Y	Y	Y	Y	Y	Y	Y
Tear meniscus height (TMH)		Y	Y	Y	Y	Y	Y	Y	Y	Y
Lipid layer thickness (LLT)		Y	Y	Y	Y	Y	Y	Y	Y	Y
Tear interferometric fringe pattern		Y	Y	Y	Y	Y	Y	Y	Y	Y
Meiboscore	Y	Y	Y	Y	Y	Y	Y	Y	Y	Y
Meibograde		Y	Y	Y	Y	Y	Y	Y	Y	Y
Partial blinking		Y	Y	Y	Y	Y	Y	Y	Y	Y
Schirmer test without anesthesia		Y	Y	Y	Y	Y	Y	Y	Y	Y
Lid margin and eyelash abnormalities		Y	Y	Y	Y	Y	Y	Y	Y	Y
Tear film break-up time (TFBUT)#	Y	Y	Y	Y	Y	Y	Y#	Y	Y	Y#
Corneal staining (Modified Oxford grading)		Y	Y	Y	Y	Y	Y	Y	Y	Y
Conjunctival staining (Modified Oxford grading)		Y	Y	Y	Y	Y	Y	Y	Y	Y
Lid wiper epitheliopathy (LWE)		Y	Y	Y	Y	Y	Y	Y	Y	Y
Meibomian gland expressibility	Y	Y	Y	Y	Y	Y	Y	Y	Y	Y
Meibomian glands yielding liquid secretion (MGYLS)		Y	Y	Y	Y	Y	Y	Y	Y	Y
**SAMPLE COLLECTION**										
Blood		Y								
Conjunctival microbiome		Y				Y				Y
Tear specimen by Schirmer test strips		Y	Y	Y	Y	Y	Y	Y	Y	Y
Meibum 1^st^ by swab*		Y	Y	Y	Y					
Meibum 2^nd^ by swab^^^		Y				Y				Y
**SAFETY**										
Procedure-related pain (VAS)*		Y	Y	Y	Y					
Visual acuity	Y	Y	Y	Y	Y	Y	Y	Y	Y	Y
Intraocular pressure	Y	Y	Y	Y	Y	Y	Y	Y	Y	Y
Adverse event and safety checking		Y	Y	Y	Y	Y	Y	Y	Y	Y

Abbreviation: V: visit; M: month; Y: scheduled; #: Primary outcome; *: For Group A- VTP subjects after receiving the VTP session immediately, and Group B- IPL + MGX subjects after MGX immediately (OD & OS, Lower Eyelid); ^: For all subjects during quality of expressed meibum examination (OD & OS, Lower Eyelid)

No independent Data Monitoring Committee was convened because the interventions are classified as carrying minimal or limited risk under institutional guidelines. Safety monitoring was performed by the study team and the principal investigator. Predefined adverse event definitions and severity grading, based on the SUN and LOCS II systems, were included as study SOP. Subjects experiencing serious or procedure-related adverse events, including microbial keratitis, or unexpected severe ocular surface complications would be withdrawn from the study but invited to continue the remaining study visits.

Subjects had the right to withdraw their consent at any stage. If they developed microbial or marginal keratitis, serious adverse events potentially attributable to VTP or IPL + MGX, or developed worsening or progression of MGD, they would be withdrawn from the study and managed according to the standard of practice.

### Statistical analysis

The REDCap (Research Electronic Data Capture) version 14 (Vanderbilt University, Tennessee, USA) web application is used to set up database, monitor data storage, and provide quality control. The intention-to-treat analysis (ITT) will be applied using Prism version 10 (GraphPad Software, California, USA) and IBM SPSS Statistics 29 (New York, USA). The data analysis and reporting will follow the CONSORT guideline for RCT [41].

Descriptive statistics of subjects’ demographics, baseline characteristics, clinical outcome measures, and adverse events will be reported. Continuous variables will be presented as mean ± SD, or median with interquartile range (IQR) unless otherwise stated. The quantitative variables at baseline will be analyzed by the Kolmogorov-Smirnov test with Lilliefors corrections for confirmation of normal distribution. The parametric and nonparametric methods will be selected accordingly. The homogeneity between the groups will be analyzed by Analysis of Variance (ANOVA) or the Kruskal-Wallis H test. Categorical data will be presented as frequencies and percentages. Primary analysis will follow the intention-to-treat principle (ITT). The multiple imputation (MI) will be applied to handle any missing data. 5 datasets on MI will be generated, and the pooling result will be used for the final analysis. Generalized estimating equations (GEE) will be used to analyze repeated measures at baseline and specified follow-up intervals. Selected baseline variables will be included as covariates. Subgroup analyses by predefined minimization covariate factors, sex, age, or MGD severity, will be performed. P value <0.05 will be considered statistically significant unless stated otherwise.

### Ethics approval

This study complies with the Declaration of Helsinki and the ICH-GCP [42]. The study was reviewed and approved by the Joint CUHK-NTEC Clinical Research Ethics Committee (CREC) (no.: 2021.271-T), the Kowloon Central/ Kowloon East Cluster CREC (no.: 21–0127/FR-1), and the CUHK Medical Centre CREC (no.: CREC-202209). It was registered on ClinicalTrials.gov (Clinical Trial Study ID: NCT05577910, first submitted on 2022-09-27). All subjects were required to sign the consent form before participating in the study. All subjects need to state whether they agree or disagree with the collection and use of subject data and biological specimens. Both paper form consent or e-consent would be applied. This study would not recruit minors. Participants did not receive financial compensation but were reimbursed for transportation. For any important protocol modifications, the amendment would be sent to CREC for further approval and updated on the trial registries. The authorship eligibility follows the definition of the role of authors and contributors from the International Committee of Medical Journal Editors (ICMJE).

### Data management and dissemination

All clinical data were entered by designated staff onto REDCap platform and stored in pseudonymized form. Each subject has the right to acquire personal data and publicly reported results, and to request for destruction of records and results of all study investigations. As MGD is a non-life-threatening condition, no Data Monitoring Committees (DCM) was required in this study. The results of this clinical trial will be published in academic journals as level I evidence for clinical practices. The research team will present the findings at academic seminars or conferences, both locally and internationally. Relevant results will also be shared with the general public in possible public education seminars and/or press releases.

## Results and discussion

### Results

From October 2022 to March 2024, a total of 926 subjects were screened. 374 eligible subjects were recruited and subsequently randomized into 3 groups as Fig 1 (Group A- VTP: n = 125; Group B- IPL + MGX: n = 126; Group C- EW: n = 123).

372 subjects were included in the final analysis (2 subjects were excluded during washout period after receiving dermatological treatment in the periocular region or developing possible allergic reaction to the topical lubricants). Baseline characteristics by treatment groups are shown in Table 2. The mean age was 54.4 ± 14.7 years. 283 (76%) out of 372 subjects were females. At baseline, the mean TFBUT was 3.1 ± 1.7 seconds. The other key demographic and baseline characteristics of the subjects are summarized in Table 2.

**Table 2:** Baseline characteristics of subjects.

	Overall (n = 372)		Group A: VTP (n = 124)		Group B: IPL + MGX (n = 126)		Group C: EW (n = 122)	
	Mean	SD	Mean	SD	Mean	SD	Mean	SD
**Age, y**	54.4	14.72	54.29	14.22	54.22	14.31	54.7	15.73
**Sex, No. of female (n, %)**	283	(76.07)	94	(75.81)	95	(75.4)	94	(77.04)
**Ethnic group (n, %)**								
Asian	372	(100)	124	(100)	126	(100)	122	(100)
**OSDI**	33.96	20.31	33.25	21.65	35.9	19.09	32.68	20.13
**SANDE**	59.28	25.76	57.24	27.16	61.56	24	59.02	26.07
**SPEED**	12.08	5.48	12.13	5.97	12.37	5.25	11.75	5.19
**DEQ-5**	11.42	4.32	11.32	4.54	11.82	4.37	11.12	4.04
**OCI-C**	38.61	11.07	39.14	11.7	39.14	9.98	37.49	11.48
**TFBUT, sec.**	3.10	1.65	2.98	1.56	3.17	1.83	3.15	1.55
**Oxford conjunctival score**	0.52	0.85	0.51	0.81	0.47	0.90	0.58	0.84
**Oxford corneal score**	0.48	0.74	0.49	0.69	0.47	0.80	0.50	0.73
**Lid wiper epitheliopathy**	2.13	1.81	2.1	1.78	2.16	1.74	2.12	1.93
**QEM sum**	20.89	10.87	21.76	10.56	19.59	10.8	21.37	11.23
**MGYLS**	3.26	4.00	3.65	4.13	2.70	3.63	3.45	4.12
**LLT max, nm**	84.41	17.12	83.93	16.18	84.68	17.67	84.63	17.62
**LLT ave, nm**	70.11	21.18	70	21.4	70.95	21.33	69.34	20.96
**LLT min, nm**	57.89	22.71	57.16	22.61	58.74	23.83	57.77	21.77
**Partial blink rate (%)**	-	(56.33)	-	(54.59)		(56.52)		(57.83)
**tear interferometric fringe pattern**	2.46	1.31	2.52	1.33	2.46	1.31	2.39	1.31
**Meiboscore l.l.**	1.51	0.71	1.57	0.74	1.49	0.68	1.48	0.71
**Meiboscore u.l.**	1.47	0.7	1.51	0.68	1.52	0.71	1.39	0.71
**Meibograde u.l.**	4.15	1.67	4.20	1.65	4.28	1.66	3.97	1.69
**Meibograde l.l.**	3.9	1.67	4.00	1.72	3.85	1.60	3.84	1.71
**NIKBUT ave, sec.**	11.45	5.23	11.07	5.19	11.52	5.25	11.77	5.28
**NIKBUT first, sec.**	8.53	4.90	8.56	4.90	8.41	5.05	8.62	4.76
**bulbar conjunctival hyperaemia**	1.15	0.45	1.16	0.44	1.16	0.47	1.15	0.45
**TMH, mm**	0.34	0.14	0.33	0.13	0.34	0.13	0.35	0.17
**ST, mm**	9.81	8.05	9.03	7.23	10.89	9.41	9.48	7.21

Abbreviations: SD: standard deviation; OSDI: the Ocular Surface Disease Index; SANDE: the Symptom Assessment iN Dry Eye; SPEED: Standard Patient Evaluation of Eye Dryness; DEQ-5: Dry Eye Questionnaire; OCI-C: Ocular Comfort Index in Chinese; TFBUT: tear film break-up time; QEM: quality of expressed meibum; MGYLS: meibomian glands yielding liquid secretion;LLT: lipid layer thickness; avg.: average; min.: minimum; max.: maximum; u.l.: upper lid; l.l.: lower lid; NIKBUT: non-invasive keratograph break-up time; TMH: lower eyelid tear; meniscus height; ST: Schirmer test

The study team would follow the above study protocol closely to provide a comprehensive assessment of the efficacy and safety of VTP, IPL + MGX, and EW over the 15-month study period.

## Discussion

The reported prevalence and incidence of MGD are notably high across various Asia Pacific regions and countries [43–45]. Within the spectrum of MGD, mild to moderate severity constitutes the majority. Untreated mild to moderate MGD might progress and worsening of ocular surface disease might develop [46,47]. At present, there is a lack of high-quality evidence comparing the head-to-head efficacy of VTP, IPL + MGX, and EW [48]. Therefore, comparing the efficacy of these three physical interventions, as investigated in this study, is critical to fill the management gap of MGD.

This is the first large-scale RCT with long-term follow-up data to shed important insight to inform clinical practice. To ensure reliability and explore potential biomarkers on MGD, an exhaustive panel of objective and subjective tests has been included. Importantly, investigators performing these assessments were masked to the treatment allocation.

Furthermore, detailed SOPs were designed and followed for all examinations. For instance, pipette tip was used to administer 2 µL of fluorescein sodium onto the lower lid conjunctiva sac and the TFBUT was immediately measured by calculating the average of three consecutive measurements using a smartphone-based timer of each eye. And a standardized sequence of tests and procedures, from least to most invasive, was administered at every visit. These standardized approaches ensured the consistency and validity of the primary outcome and related findings. In this study, specimens of meibum, swabs and tears were also collected. Molecular analyses of these samples may provide mechanistic insights behind these interventions.

The baseline characteristics of the enrolled patients were consistent with the predefined inclusion and exclusion criteria of mild to moderate MGD. The baseline results confirmed the feasibility of the subjective and objective outcome measures in the study protocol. The same order from least to most invasive investigations would be conducted at each visit. Moreover, all outcome measures at baseline directly reflected the characteristics of MGD of mild to moderate severity in this territory-wide Hong Kong cohort. The female predominance of participants is consistent with known MGD epidemiology among other Asian cohorts.

## Conclusion

The study protocol above aims to provide level I evidence of managing mild to moderate MGD by the three physical treatments.

## Supporting information

S1 File The SPIRIT (Standard Protocol Items: Recommendations for Interventional Trials) checklist. (DOC)

S2 File The CONSORT (CONsolidated Standards Of Reporting Trials) checklist. (DOCX)

S3 File Research protocol approved by the ethics committee. This is the research protocol reviewed by the Joint CUHK-NTEC Clinical Research Ethics Committee (CREC) (no.: 2021.271-T). It is the version 4 updated on 6 Mar 2024 and approved on 6 April 2024.
(DOCX)
