# Supplementary material for: Safety and efficacy of Vectored thermal pulsation, Intense pulsed light, and Eyelid Warm compress therapies (VIEW) for meibomian gland dysfunction: Study design and baseline characteristics of a randomized controlled trial
Source: PLoS One. 2026 Apr 2;21(4):e0342421. doi: 10.1371/journal.pone.0342421 (PMC13046148; doi:10.1371/journal.pone.0342421)
Supplement: S3 File — This is the research protocol reviewed by the Joint CUHK-NTEC Clinical Research Ethics Committee (CREC) (no.: 2021.271-T). It is the version 4 updated on 6 Mar 2024 and approved on 6 April 2024. (DOCX) [file pone.0342421.s003.docx]

**Research Protocol**

**V**ectored Thermal Pulsation, **I**ntense Pulsed Light, and **E**yelid **W**arm Compress (VIEW) therapies for meibomian gland dysfunction - a multicentre, randomized, assessor-masked, active-controlled clinical trial

This is the research protocol reviewed by the Joint CUHK-NTEC Clinical Research Ethics Committee (CREC) (no.: 2021.271-T). It is the version 4 updated on 6 Mar 2024 and approved on 6 April 2024.

# Introduction

Dry eye disease (DED) is an emerging, under-recognized and under-treated epidemic of the 21st century. It is one of the commonest reasons seeking eye consultation worldwide. Depending on the diagnostic criteria, geographic location and population concerned, prevalence of DED ranges from 30 to over 50% in the Asia Pacific regions compared to around 5-10% in the United States [1-2]. Up to 90% of DED is closely associated with meibomian gland dysfunction (MGD),[3] a chronic and progressive condition characterized by terminal duct obstruction, qualitative and/or quantitative changes in the glandular secretions (meibum) which causes tear instability by increasing tear evaporation and subsequently increased tear osmolarity, ocular surface inflammation, epithelial damage and ocular surface disease. Studies suggest that MGD affects between 5-20 % of Caucasians and over 60% of Asians populations.[4-5]

Conventional treatments for MGD including self-administered eyelid hygiene, eyelid warm compress therapy (EW), artificial tears, including lipid‐containing lubricants are limited in their efficacies for moderate to advanced disease.[6] Prescription medications (topical steroids, topical and oral antibiotics, topical immunomodulatory agents e.g. cyclosporine and oral omega-3 essential fatty acids) have demonstrated efficacies in improving symptoms and signs of MGD;[7-8] however side-effects including preservative-related adverse events, development of antibiotic resistance, cost, accessibility, off‐label use, and the need for ongoing treatments often limit their long‐term use.[9-10]

Despite the described range of available options, management for MGDs is often considered unsatisfactory and frustrating by clinicians and patients. Compliance to long-term, home‐based self-administered therapies is known to be suboptimal [8-10] while practitioner-administered treatment including meibomian gland expression (MGX) provides transient relief.[11-12]

Intense pulsed light (IPL) therapy is widely used in cosmetic dermatology as well as therapeutically for a wide range of skin conditions with favourable efficacy and tolerability. Concurrent MGD improvements were observed serendipitously in patients undergoing IPL for rosacea.[13] With growing interest in combining IPL+MGX as practitioner-administered physical therapy for MGD, recent review and meta-analysis showed its effectiveness and safety while calling to investigate its effect beyond 6 months after the last IPL treatment [14-16].

Vectored thermal pulsation (VTP) is approved by FDA as another practitioner-administered physical therapy for MGD. The device covers both the cutaneous and mucosal surfaces of the eyelids; the rear portion of the device provides heat to the MG, and the front portion gives mechanical stimulation to the eyelid skin. It evacuates the MG of the upper and lower eyelids simultaneously with minimal discomfort while protecting the cornea, rendering the experience for patients generally favorable. Recent meta-analysis showed that a single 12-minute VTP was more efficacious than EW in treating MGD [17].

Knowledge gaps

Level I evidence comparing efficacies between two promising practitioner-administered therapies VTP and multi-session IPL+MGX with standard self-administered twice-daily EW for MGD is currently lacking. The onset and offset of therapeutic effects, time course of multi-session IPL+MGX, single-session VTP or twice-daily EW on MGD up to 15-month post-treatment initiation or 12-month post last session of IPL have not been studied either in an RCT setting. These important gaps will be addressed in this application.

# Aims and hypotheses to be tested

Primary Objective

To compare the efficacy and safety of 1-session vectored thermal pulsation (VTP) or 4-session intense pulsed light and meibomian gland expression (IPL+MGX) with twice-daily eyelid warm compress (EW) therapy for meibomian gland dysfunction (MGD)

Secondary Objectives

1. To compare the course of MGD among groups over 15 months (12-month after final IPL+MGX)
2. To identify factors predicting responses and compliance to therapies

Hypotheses

1. Both 1-session VTP and 4-session IPL+MGX are more efficacious than twice-daily EW in improving MGD
2. MGD improves earlier after VTP
3. MGD improvement lasts longer after 4-session IPL+MGX

# Plan of investigation

## Subjects

A total of 360 patients with symptomatic MGD will be recruited from the participating hospitals coordinated by the Chinese University of Hong Kong (CUHK) Research Clinic, the CUHK Eye Centre (CUHKEC), Department of Ophthalmology and Visual Science, Faculty of Medicine, The CUHK. Open recruitment will be made possible by the use of online channels, as well as promotional flyers and QR codes to be distributed at the participating hospitals. We adopt some of the inclusion and exclusion criteria of recent RCT on MGD [18-19].

Inclusion criteria include:

1. ≥18 years of age with no upper age limit;
2. Providing informed consent, agree to attend follow-up visits and comply to treatment regimen
3. Symptomatic dry eye TFBUT ≤ 5 seconds and OSDI ≥ 13[20];
4. Mild to moderate (level 3-4) MGD on at least one eye[21];
5. Fitzpatrick skin type 1-4.

Exclusion criteria are:

1. Contact lens wear 3 months before or during the study period;
2. Use of topical (including anti-glaucomatous, cyclosporin, antibiotics) or systemic medication known to affect (worsen or improve) MGD 3 months before or during the study period;
3. Major systemic (e.g. Sjogren’s syndrome), dermatologic (e.g. Rosacea) known to affect MGD or ocular conditions (including thyroid eye disease, recurrent conjunctivitis, ocular allergies);
4. Ocular procedures (excluding uncomplicated cataract operation) 3 months before and any ocular procedure during the study period;
5. History of vision correction surgery or plan to undergo the procedure during the study period;
6. Dermatological treatment (including chemical peeling, laser, IPL or energy device in the periocular and facial region) 6 months before or during the study period;
7. Contraindications to IPL therapy (including recent sun-burn, photosensitivity, active or pigmented skin lesions, cancer, implants, tattoos, semi-permanent makeup in the periocular area);
8. Contraindications to VTP therapy (ocular surgery, ocular injury, ocular herpes of eye or eyelid, and ocular inflammation 3 months before the study; active ocular infection, etc.);
9. Women who are pregnant, nursing, planning pregnancy, or of childbearing potential not using a reliable method of contraception.

## Methods

All measurements are conducted in accordance with the recommendations of the TFOS DEWS II Diagnostic Methodology subcommittee [22].

Participants will be assessed in a designated room at CUHK Eye Centre or CUHK Medical Centre Eye Centre with temperature of 20.3±0.5°C and relative humidity of 63±7% by the same study personnel (research assistants) masked to the treatment assignment. To minimise the impact on subsequent tests, measurements are performed in ascending order of invasiveness and are summarised in Table. The following will be measured or conducted before each treatment or follow-up visit.

Questionnaires (Traditional Chinese version)

Demographic and baseline characteristics, chronic condition, eye disease, surgery & hospitalization history, smoking & alcohol use history, life conditions, and work productivity, etc., are collected via standard questionnaires when subjects are recruited. The 12-item symptom frequency-based questionnaire Ocular Surface Disease Index **(OSDI)**, Standard Patient Evaluation of Eye Dryness **(SPEED)**, 2-item frequency- and severity-based visual analog scale Symptom Assessment iN Dry Eye **(SANDE)**, Ocular comfort index **(OCI),** and Dry eye questionnaire **(DEQ-5)** are self-administered at each study visit.

Visual acuity

Visual acuity of the participant is examined by a standard visual acuity chart as safety outcome measures.

Intraocular pressure

The intraocular pressure, the fluid pressure of the eye, is assessed by a non-contact tonometer as the safety outcome measure.

Keratograph 5M (Oculus, Wetzlar, Germany)

**Non-invasive keratograph break-up time (NIKBUT)** is measured using automated detection of first break-up, while the subject maintains fixation and is requested to refrain from blinking. Three breakup time readings are averaged in each case.

**Bulbar conjunctival hyperaemia** is evaluated according to the proprietary JENVIS grading scale from 0 to 4.

The lower **tear meniscus height (TMH)** is assessed using high magnification pre-calibrated digital imaging, and three measurements near the centre of the lower meniscus are averaged.

Lipiview II

**Lipid layer thickness (LLT)** of the tear film as determined with LipiView II (TearScience, Morrisville, NC).

**Tear interferometric fringe pattern** is graded according to the modified Guillon-Keeler system: grade 1, open meshwork; grade 2, closed meshwork; grade 3, wave or flow; grade 4, amorphous; grade 5, colored fringes; grade 0, non-continuous layer (non-visible or abnormal colored fringes).

**Infrared meibography** is taken with the superior and inferior eyelids everted in turn. From the captured image, the proportion of meibomian glands visible within the tarsal area is graded according to the four-point meiboscore (0-3) by Arita et al. Morphological changes of meibomian glands is assessed by the ‘meibograde’ method evaluating gland distortion, gland shortening and gland dropout. Each of these characteristics is graded from 0-3, like the meiboscore, and then they are summed to give a total score of 0-9 per eyelid where a higher score represents a higher percentage of gland loss.[25]

**Partial blinking** is reported as % recorded by the Lipiview II.

Schirmer’s test (ST)

**Schirmer’s test (ST)** will be performed without anesthesia for 5 mins. The strips will be collected in sterile centrifuge tubes respectively for laboratory tests of biomarkers.

Slit-lamp biomicroscopy (to be performed by masked follow-up investigators)

**Lid margin and eyelash abnormalities**, including lid margin thickening, rounding, notching, foaming, telangiectasia, meibomian gland capping, staphylococcal lash crusting, seborrheic lash crusting, Demodex lash cylindrical collarettes, madarosis, poliosis, and trichiasis, are graded based on a four-point scale: grade 0, absent; grade 1, mild; grade 2, moderate; grade 3, severe.

Sodium fluorescein dyes are applied using the recommended technique described [21].

**Tear film break-up time (TFBUT)**, defined as the time to initial breakup of the tear film after a blink is measured three times.

**Corneal and conjunctival staining** score due to epithelial desiccation is documented according to the modified Oxford grading scheme.

**Lid wiper epitheliopathy (LWE)** is evaluated relative to Korb’s grading.

**Expressibility** of the inferior eyelid meibomian glands is assessed with the Meibomian Gland Evaluator (TearScience, North Carolina, USA) applied just inferior to the nasal, central, and temporal aspects of the eyelid margin. The proportion of meibomian orifices yielding lipid secretions is graded on a five-point scale: 0, more than 75%; 1, 50% to 75%; 2, 25% to 50%; 3, less than 25%; 4, none.

The meibomian glands yielding liquid secretion (MGYLS) is the total number of glands which yielding lipid secretions at the nasal, central, and temporal aspects of the eyelid margin (5 glands each part).

**Quality of expressed meibum** is graded on a four-point scale: grade 0, no secretion; grade 1, inspissated (semi-solid, toothpaste-like consistency); grade 2, colored/ cloudy liquid; grade 3, clear liquid oil. The sum scores of 15 glands will be between 0 and 45.

Dilated fundus examination, anterior chamber activities, lens opacity based on lens opacities classification system II (LOCS II), and iris defect/transillumination will be conducted as safety outcomes.

Conjunctival swab sampling

Sterile, polyester swabs will be applied for conjunctival swab sampling on the inferior fornix of the conjunctiva in each eye. The swabs will be placed in microcentrifuge tubes. Extraction will be performed by laboratory staff, then the sample will be transferred to a -80 °C laboratory freezer for long-term storage before laboratory tests.

Blood specimens collection

Participant's blood specimens will be collected at the baseline visit and pre-treated for further laboratory tests. After genomic DNA extraction from venous blood, Single-nucleotide polymorphism (SNP) will be selected, sequenced and compared between treatment groups for pharmacogenomic analyses.

Expressed meibum sampling

Sterile, polyester swabs will be applied for collection of expressed meibum on the nasal, central, and temporal aspects of the eyelid margin in each eye during the examination of expressibility and quality of expressed meibum.

The expressed meibum by compression forceps after MGX treatment procedure will be collected among the IPL+MGX group subjects.

The swabs will be placed in microcentrifuge tubes. Extraction will be performed by laboratory staff, then the sample will be transferred to a -80 °C laboratory freezer for long-term storage before laboratory tests.

Vectored thermal pulsation (VTP) therapy

VTP (LipiFlow® Thermal Pulsation System, Johnson & Johnson, USA) is an automated thermal pulsation system that is designed to heat and simultaneously evacuate the meibomian gland contents. It is performed by the unmasked treating investigators as described by the equipment manufacturer. In brief, one to two drops of topical anesthesia are applied prior to the bilateral application of the activators. The inner portion of the activator applies a constant temperature of 42.5°C to the tarsal conjunctiva of upper and lower eyelids. Simultaneously, the outer portion of the activator applies directional, pulsatile pressure to the external eyelid surfaces (maximum 6 psi) for the 12-minute treatment cycle. Subjects will feel warmth and pressure on their eyelids during the 12-minute bilateral procedure. All subjects are examined at the slit lamp after the procedure. A procedure related pain score by Visual Analogue Scale (VAS) is obtained by the treating investigator immediately.

Intense pulsed light with meibomian gland expression (IPL+MGX) therapy

IPL is delivered using the proprietary “dry eye mode” setting of the Lumenis® M22TM system (skin type settings 1–4 and mode A–F) per the Toyos protocol (13). Pulse intensity (11-14 J/cm2) is inversely related to the Fitzpatrick skin phototype of each participant and will be used for the same patient throughout the study. Adjustment is allowed due to safety (e.g. treatment related pain) and will be recorded. Clear conducting gel will be applied to the inferior, lateral, and medial aspects of the goggles, as per manufacturer recommendations. IPL will be delivered to four overlapping zones inferior to each eye and a fifth pulse applied temporally adjacent to the lateral canthus four times (month 0,1,2,3). Both eyelids are closed and sealed with IPL-Aid disposable eye shields (Honeywell Safety Products, Smithfield, RI). Safety goggles are worn by the unmasked treating investigators and trial coordinator present during IPL. Meibomian gland expression is immediately performed on both upper and lower eyelids of each eye and pain will be minimized by topical anesthetic. A procedure related pain score by VAS is obtained by the treating investigator immediately. Visual acuity, slit lamp examination to record any iris injury, change in lens opacity by LOCSIII, and external examination on periocular skin, eye lash and eyebrow hair injuries before and after IPL and at each study visit. Each session of IPL and MGX takes about 20 minutes.

Both VTP and IPL therapies are not available and have not been adopted in public hospitals under the Hospital Authority.

Eyelid Warm Compress Therapy (EW)

EW using warm wet towel to each eye is performed twice daily for 10 mins [9-11]. A treatment diary will be given for patients randomized to EW for daily recording which will be reviewed and recorded by the unmasked study coordinator at each study visit.

## Study design

This is a prospective, multicentre, randomized, assessor-masked, 3-arm (1:1:1), active-controlled trial of 360 subjects with meibomian gland dysfunction contributing one study eye. For subjects with both eyes eligible, the eye with the most meibomian gland dropout (meiboscore), fewest expressible meibomian glands, thinnest lipid layer, or lowest TFBUT values (in this order) will be selected as the study eye.

Enrolled patients will be randomized into one of the following groups, 1 month after recruitment during the 15-month study period receiving bilateral treatment of:

Group A: 1-session VTP at month 0

Group B: 4-session IPL+MGX at month 0,1,2,3

Group C: twice daily EW for 15 months

The study will be conducted in accordance with the tenets of the Declaration of Helsinki, and the protocol will be sent for approval by the research ethics review committee of each participating hospital. All participants will provide written informed consent before participation. Study will be registered at ClinicalTrials.gov prior to recruitment. The baseline, follow-up assessments and study procedures will be conducted as described above. Participants will be given contact of the study team to report adverse events at any time, which will also be actively sought at each follow-up visit. Additional follow-up appointment will be provided as and when required.

Recruitment, baseline and follow-up assessment and tear substitutes

Patients will be washed out for 1 month and maintained on one single topical lubricant to be used as frequently as needed from recruitment to study exit (total 16 months). Cool Eye eye drops- hypromellose (3 mg/ml) containing 0.0002 ml of 50% Benzalkonium Chloride solution as preservative will be provided by the research team.

Clinical evaluations are done according to the recommendations of the TFOS DEWS II diagnostic methodology subcommittee at month -1 (recruitment), 0 (baseline), 1, 2, 3, 4, 6, 9, 12, 15 (total 10 visits). Examination is conducted on both eyes starting on the study eye. To minimize the impact on ocular surface and tear film physiology, tests are done from least to most invasive at each follow-up and/or prior to each treatment.

Randomization, masking of assessors

Randomization will be carried out by a computer-generated minimization program. Minimization is a dynamic process to reduce the imbalance between trial arms with respect to a range of predefined prognostic variables, and a randomisation schedule is therefore not drawn up in advance. A form describing the baseline characteristics of each subject according to these minimisation criteria: gender (female, male), age (<45, >45), worst meiboscore (0 vs 1 vs 2 vs 3) from the study eye (lower lid) and referring centres will be filled at recruitment (month-1), returned to Centre for Clinical Research & Biostatistics, CUHK on enrolment. Treatment allocation will be sent to the unmasked trial coordinator for arrangement at baseline (month 0).

IPL or VTP is given by **unmasked treating investigators** not involved in data collection.

**Follow-up investigators** collecting the data are masked to participants’ treatment assignment. This information can be disclosed upon request after the completion of the study.

**Unmasked trial coordinator** will ensure masking by reminding and accompanying each patient before and during visit. Treatment related complications will be evaluated by all participants in a standard datasheet regardless of group assignment. Follow-up investigators will be asked if they know each participant’s group assignment at each visit and why.

Escape rule/withdrawal

Subjects recruited from HA can withdraw their consents at any stage but will be invited to continue follow-up at CUHKEC. They will be managed as per standard of care by referring ophthalmologist. Subject will leave study protocol and be terminated from the study but will be followed and assessed as a study patient if they develop any of the following complications:

1. microbial or marginal keratitis
2. Serious Adverse Event (SAE) potentially attributable to VTP or IPL+MGX

Subjects who develop worsening/progression of their MGD will be managed as per medical standard by their referring ophthalmologist by topical/systemic antibiotics, meibomian gland expression which will be documented as protocol deviation.

## Data processing and analysis

Trial biostatistician will oversee data acquisition, processing and statistical analysis.

Record keeping, confidentiality and monitoring

A unique study number will be assigned to each recruited subject for all study-related documents. The log containing study number to identifiable information will be kept in a locked cabin at the clinical trial office at CUHKEC. All trial-related documents will be maintained by the unmasked trial coordinator to ensure data are accurate, complete, and verifiable from case report forms. Data entry will be cross-checked by a designated technician.

**Primary outcome**: Tear film breakup time (TFBUT) at month 6, month 15

**Secondary outcomes**: Serial measurements of MG, tear-film related parameters, intraocular pressure, compliance to EW, factors associated with MGD outcomes and treatment related complications.

Sample size calculation

Based on Arita et al for IPL+MGX[23] and Lane et al for VTP[24], the estimate mean differences in changes of TFBUT are 3.3 for IPL+MGX and 1.9 for VTP, the pool estimates of SDs are 1.88 for IPL+MGX and 4.04 for VTP. Given a 2.5% type I error (Bonferroni correction) and assuming an average SD of 3, a minimum sample of 88 patients per group can detect a significance difference in change of TFBUT between groups. By assuming a 35% of drop-out over an extended study period (16 months from recruitment), 120 subjects per group are required. This sample size is powered to detect differences between VTP and EW or between IPL and EW given the estimates of EW from Lane et al.

Statistical analysis

The intention-to-treat principle will be applied during the data analysis using Graph Pad Prism version 8.01 (California, USA) and IBM SPSS version 24 (New York, USA) and reported according to CONSORT guidelines for RCT. Categorical data at baseline are analysed using chi-squared or Fisher’s exact tests. Data are presented as mean± SD, or median (IQR) unless otherwise stated. Study outcomes will be compared between groups using pair-wise t-test. Continuity-adjusted chi-squared testing will be applied to compare proportion of patients between treatment arms on secondary outcome. Further comparisons of the primary and secondary outcomes between treatment arms, at baseline and pre-set intervals will be made using risk ratio (RR) for dichotomous data and mean difference for continuous data with 95% confidence interval. Appropriate (logistic or linear) regression models, adjusting for minimisation and outcome variables at baseline will be computed for prognostic factors. Multivariate analyses will be conducted to control with baseline measurements, covariates, and repeated measurements. Correlations between parametric and nonparametric data are assessed with Pearson product-moment correlation or Spearman rank order correlation, respectively. The proportions of eyes developing complications during and after the procedures are compared with Chi-square test. All tests are two-tailed, and p<0.05 is considered significant.

# Ethical concerns

Patient data would be handled with utmost care not to breach patient's privacy in any form. The data would be stored in secure cabinets and/or computers which would be password-protected. To protect patient privacy, all research data would be handled in line with HA / Hospital’s policy in handling / storage / destruction of patients’ medical records. Electronic data would be saved in secured computer of the hospital with restricted access. USB Device would not be used for patient information nor personal data. Personal data (name, HKID, OPD / hospital numbers, address and any other personal identifiable information) would not be recorded on the project’s data sheets or electronic files. A study code would be used instead. The document of electronic file containing the linkage information between the study code and the identity of the patient would not contain any other information and would be kept separate from the study data files or data sheets with the same stringent security as the medical record. Any documents or electronic files containing personal identifiable information would be considered as part of the medical record and would be dealt with the same stringent regulations of security according to the hospital policies. All the investigators would be responsible for data handling and protection.

# Key references

1. Nelson JD, Craig JP, Akpek EK, Azar DT, Belmonte C, Bron AJ, et al. TFOS DEWS II introduction. Ocul Surf 2017;15:269–75.
2. Schein OD, Munoz B, Tielsch JM, Bandeen-Roche K, West S. Prevalence of dry eye among the elderly. American journal of ophthalmology. 1997;124(6):723-8.
3. Chan TCY, **Chow SSW, Wan KHN**, Yuen HKL. Update on the association between dry eye disease and meibomian gland dysfunction Hong Kong Med J. 2019;25(1):38-47
4. Schaumberg DA, Nichols JJ, Papas EB, Tong L, Uchino M, Nichols KK. The international workshop on meibomian gland dysfunction: report of the subcommittee on the epidemiology of, and associated risk factors for, MGD. Investig Ophthalmol Vis Sci 2011;52:1994–2005
5. Nelson JD, Shimazaki J, Benitez-del-Castillo JM, et al. The international workshop on meibomian gland dysfunction: report of the definition and classification subcommittee. Invest Ophthalmol Vis Sci. 2011;52(4):1930-7
6. Thode AR, Latkany RA. Current and emerging therapeutic strategies for the treatment of meibomian gland dysfunction (MGD). Drugs 2015;75:1177–85.
7. Wladis EJ, Bradley EA, Bilyk JR, Yen MT, Mawn LA. Oral Antibiotics for Meibomian Gland-Related Ocular Surface Disease: A Report by the American Academy of Ophthalmology. Ophthalmology. 2016 Mar;123(3):492-6
8. Dry Eye Assessment and Management Study Research Group, Asbell PA, Maguire MG, Pistilli M, Ying G, Szczotka-Flynn LB, Hardten DR, Lin MC, Shtein RM. n-3 Fatty Acid Supplementation for the Treatment of Dry Eye Disease. N Engl J Med. 2018 May 3;378(18):1681-1690.
9. Lam PY, **Shih KC,** Fong PY, Chan TCY, Ng ALK, Jhanji V, Tong L. A Review on Evidence-Based Treatments for Meibomian Gland Dysfunction Eye Contact Lens. 2020 Jan;46(1):3-16.
10. Foulks GN, Nichols KK, Bron AJ, Holland EJ, McDonald MB, Daniel Nelson J. Improving awareness, identification, and management of meibomian gland dysfunction. Ophthalmology. 2012 Oct;119(10 Suppl):S1-12.
11. Sabeti S, Kheirkhah A, Yin J, Dana R. Management of meibomian gland dysfunction: a review. Surv Ophthalmol. Mar-Apr 2020;65(2):205-217.
12. Aketa N, Shinzawa M, Kawashima M, Dogru M, Okamoto S, Tsubota K, Shimazaki J. Efficacy of Plate Expression of Meibum on Tear Function and Ocular Surface Findings in Meibomian Gland Disease. Eye Contact Lens. 2019 Jan;45(1):19-22.
13. Toyos R, McGill W, Briscoe D. Intense pulsed light treatment for dry eye disease due to meibomian gland dysfunction; a 3-year retrospective study. Photomed Laser Surg 2015;33:41–6.
14. Wladis EJ, Aakalu VK, Foster JA, Freitag SK, Sobel RK, Tao JP, Yen MT. Intense Pulsed Light for Meibomian Gland Disease: A Report by the American Academy of Ophthalmology. Ophthalmology. 2020 Sep;127(9):1227-1233.
15. Tashbayev B, Yazdani M, Arita R, Fineide F, Utheim TP. Intense pulsed light treatment in meibomian gland dysfunction: A concise review. Ocul Surf. 2020 Jul 3;18(4):583-594.
16. Leng, X., Shi, M., Liu, X. et al. Intense pulsed light for meibomian gland dysfunction: a systematic review and meta-analysis. Graefes Arch Clin Exp Ophthalmol 2020 https://doi.org/10.1007/s00417-020-04834-1
17. Pang SP, Chen YT, Tam KW, Lin IC, Loh EW. Efficacy of Vectored Thermal Pulsation and Warm Compress Treatments in Meibomian Gland Dysfunction: A Meta-Analysis of Randomized Controlled Trials, Cornea. 2019;38(6):690-697
18. Xue AL, Wang MTM, Ormonde SE, Craig JP. Randomised double-masked placebo-controlled trial of the cumulative treatment efficacy profile of intense pulsed light therapy for meibomian gland dysfunction. Ocul Surf 2020;18(2):286-297
19. Tauber J, Owen J, Bloomenstein M, Hovanesian J, Bullimore MA. Comparison of the iLUX and the LipiFlow for the Treatment of Meibomian Gland Dysfunction and Symptoms: A Randomized Clinical Trial. Clin Ophthalmol. 2020 Feb 12;14:405-418.
20. Tsubota K, Yokoi N, Watanabe H, Dogru M, Kojima T, Yamada M, Kinoshita S, Kim HM, Tchah HW, Hyon JY, Yoon KC, Seo KY, Sun X, Chen W, Liang L, Li M, Tong L, Hu FR, Puangsricharern V, Lim-Bon-Siong R, Yong TK, Liu Z, Shimazaki J, Members of The Asia Dry Eye Society. A New Perspective on Dry Eye Classification: Proposal by the Asia Dry Eye Society. Eye Contact Lens. 2020 Jan;46 Suppl 1:S2-S13.
21. Tomlinson A, Bron AJ, Korb DR, Amano S, Paugh JR, Pearce EI, Yee R, Yokoi N, Arita R, Dogru M. The international workshop on meibomian gland dysfunction: report of the diagnosis subcommittee. Invest Ophthalmol Vis Sci. 2011 Mar 30;52(4):2006-49.
22. Wolffsohn JS, Arita R, Chalmers R, et al. TFOS DEWS II Diagnostic Methodology report. Ocul Surf 2017;15:539-574.
23. Arita R, Fukuoka S, Morishige N. Therapeutic efficacy of intense pulsed light in patients with refractory meibomian gland dysfunction Ocul Surf. 2019 Jan;17(1):104-110
24. Lane SS, DuBiner HB, Epstein RJ, Ernest PH, Greiner JV, Hardten DR, Holland EJ, Lemp MA, McDonald II JE, Silbert DI, Blackie CA, Stevens CA, Bedi R. A new system, the LipiFlow, for the treatment of meibomian gland dysfunction. Cornea. 2012 Apr;31(4):396-404.
25. Call CB, Wise RJ, Hansen MR, Carter KD, Allen RC. In vivo examination of meibomian gland morphology in patients with facial nerve palsy using infrared meibography. Ophthalmic Plast Reconstr Surg. 2012;28(6):396-400.

**Study Table: Masked assessment conducted at baseline and follow-up visits**

| **Assessments/number of month** | **0** | **1** | **2** | **3** | **4** | **6** | **9** | **12** | **15** |
| --- | --- | --- | --- | --- | --- | --- | --- | --- | --- |
| Assessments performed in ascending order of invasiveness | | | | | | | | | |
| **RE-VERIFY ELIGIBILITY** |  |  |  |  |  |  |  |  |  |
| **EFFICACY (SUBJECTIVE)** |  |  |  |  |  |  |  |  |  |
| Ocular Surface Disease Index **(OSDI)** |  |  |  |  |  |  |  |  |  |
| Standardized Patient Evaluation of Eye Dryness **(SPEED)** |  |  |  |  |  |  |  |  |  |
| Symptom Assessment iN Dry Eye **(SANDE)** |  |  |  |  |  |  |  |  |  |
| Ocular comfort index **(OCI)** |  |  |  |  |  |  |  |  |  |
| Dry eye questionnaire **(DEQ-5)** |  |  |  |  |  |  |  |  |  |
| **EFFICACY (OBJECTIVE)** |  |  |  |  |  |  |  |  |  |
| Non-invasive keratograph break-up time (NIKBUT) |  |  |  |  |  |  |  |  |  |
| Conjunctival bulbar hyperaemia |  |  |  |  |  |  |  |  |  |
| Tear meniscus height (TMH) |  |  |  |  |  |  |  |  |  |
| Lipid layer thickness (LLT) |  |  |  |  |  |  |  |  |  |
| Tear interferometric fringe pattern |  |  |  |  |  |  |  |  |  |
| Meiboscore (0-3) upper lid |  |  |  |  |  |  |  |  |  |
| Meiboscore (0-3) lower lid |  |  |  |  |  |  |  |  |  |
| Meibograde (0-9) upper eyelid |  |  |  |  |  |  |  |  |  |
| Meibograde (0-9) lower eyelid |  |  |  |  |  |  |  |  |  |
| Partial blinking (%) |  |  |  |  |  |  |  |  |  |
| Schirmer’s test without anesthesia (ST) |  |  |  |  |  |  |  |  |  |
| Lid margin and eyelash abnormalities (0-3) |  |  |  |  |  |  |  |  |  |
| Tear film break-up time (TFBUT)# |  |  |  |  |  | # |  |  | # |
| Corneal staining (Modified Oxford grading) |  |  |  |  |  |  |  |  |  |
| Conjunctival staining (Modified Oxford grading) |  |  |  |  |  |  |  |  |  |
| Lid wiper epitheliopathy (LWE) |  |  |  |  |  |  |  |  |  |
| Meibomiam gland expressibility (0-5) |  |  |  |  |  |  |  |  |  |
| Meibum quality (0-45) |  |  |  |  |  |  |  |  |  |
| **SAMPLE COLLECTION** |  |  |  |  |  |  |  |  |  |
| Tears sample (Schirmer’s test strip) |  |  |  |  |  |  |  |  |  |
| Conjunctival swab |  | X | X | X |  | X | X | X |  |
| Blood specimens |  | X | X | X | X | X | X | X | X |
| Expressed meibum sample A (by forceps, IPL+MGX subjects) |  |  |  |  | X | X | X | X | X |
| Expressed meibum sample B (before treatment) |  | X | X | X |  | X | X | X |  |
| **SAFETY** |  |  |  |  |  |  |  |  |  |
| Procedure-related pain (VAS)* |  |  |  |  | X | X | X | X | X |
| Visual acuity |  |  |  |  |  |  |  |  |  |
| Intraocular pressure |  |  |  |  |  |  |  |  |  |
| Lens opacity (LOCS II) |  |  |  |  |  |  |  |  |  |
| Anterior chamber activities |  |  |  |  |  |  |  |  |  |
| Iris defect/transillumination |  |  |  |  |  |  |  |  |  |
| Dilated fundus examination |  | X | X | X |  | X | X | X |  |
| Loss of lashes, eyebrows |  |  |  |  |  |  |  |  |  |
| Facial redness |  |  |  |  |  |  |  |  |  |
| Facial swelling |  |  |  |  |  |  |  |  |  |
| Facial bruises |  |  |  |  |  |  |  |  |  |
| Facial pigmentation |  |  |  |  |  |  |  |  |  |
| **EVALUATE ASSESSOR MASKING** |  |  |  |  |  |  |  |  |  |

#primary outcome

**Study Flow Chart**

| **Continue standard topical lubricant**  **monotherapy during study period** | **Month (-1)** | **Patient recruitment and randomization (1:1:1)** | | |
| --- | --- | --- | --- | --- |
|  |  | **4-week washout with standard topical lubricant monotherapy** | | |
|  |  | **Group A** | **Group B** | **Group C** |
|  | **Month 0** | **1-session**  **vectored thermal pulsation** | **4 monthly intense pulsed light and meibomian gland expression** | **Twice-daily eyelid hygiene warm compress** |
|  | **Month 1** |  |  |  |
|  | **Month 2** |  |  |  |
|  | **Month 3** |  |  |  |
|  | **Month 4** |  |  |  |
|  |  |  |  |  |
|  | **Month 6** |  |  |  |
|  |  |  |  |  |
|  |  |  |  |  |
|  | **Month 9** |  |  |  |
|  |  |  |  |  |
|  |  |  |  |  |
|  | **Month 12** |  |  |  |
|  |  |  |  |  |
|  |  |  |  |  |
|  | **Month 15** |  |  |  |
